# 9-Butyl-9*H*-carbazole

**DOI:** 10.1107/S1600536809005583

**Published:** 2009-02-21

**Authors:** Lei Chen, Wei Cheng, Guang-Liang Song, Hong-Jun Zhu

**Affiliations:** a Department of Applied Chemistry, College of Science, Nanjing University of Technology, Nanjing 210009, People’s Republic of China

## Abstract

The title compound, C_16_H_17_N, is a carbazole derivative that has been designed and synthesized as a potential organic electronic device, such as an OLED. The tricyclic aromatic ring system is essentially planar, the two outer rings making a dihedral angle of 4.8 (1)°. No classical hydrogen bonds are observed in the crystal structure.

## Related literature

For typical bond lengths in organic structures, see: Allen *et al.* (1987 ▶); For general background and related structures, see: Yang *et al.* (2004 ▶).
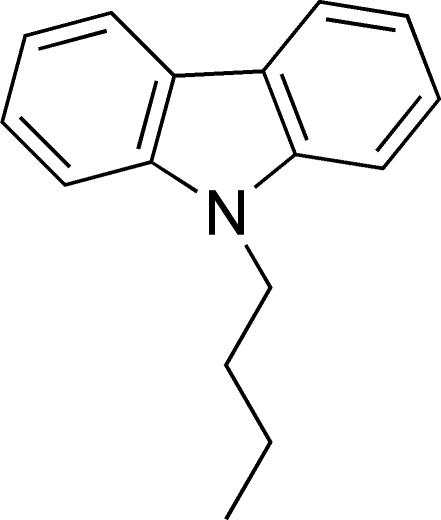


## Experimental

### 

#### Crystal data

C_16_H_17_N
*M* *_r_* = 223.31
Orthorhombic, 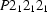

*a* = 5.544 (1) Å
*b* = 11.276 (2) Å
*c* = 20.369 (4) Å
*V* = 1273.4 (4) Å^3^
*Z* = 4
Mo *K*α radiation
μ = 0.07 mm^−1^
*T* = 298 K
0.30 × 0.20 × 0.10 mm

#### Data collection

Enraf–Nonius CAD-4 diffractometer
Absorption correction: ψ scan (North *et al.*, 1968 ▶) *T*_min_ = 0.980, *T*_max_ = 0.993
2671 measured reflections
1372 independent reflections
1500 reflections with *I* > 2σ(*I*)
*R*_int_ = 0.062
3 standard reflections every 200 reflections intensity decay: 1%

#### Refinement

*R*[*F* ^2^ > 2σ(*F* ^2^)] = 0.059
*wR*(*F* ^2^) = 0.147
*S* = 1.00
1372 reflections
154 parameters
H-atom parameters constrained
Δρ_max_ = 0.17 e Å^−3^
Δρ_min_ = −0.14 e Å^−3^

### 

Data collection: *CAD-4 EXPRESS* (Enraf–Nonius, 1994 ▶); cell refinement: *CAD-4 EXPRESS*; data reduction: *XCAD4* (Harms & Wocadlo, 1995 ▶); program(s) used to solve structure: *SHELXS97* (Sheldrick, 2008 ▶); program(s) used to refine structure: *SHELXL97* (Sheldrick, 2008 ▶); molecular graphics: *SHELXTL* (Sheldrick, 2008 ▶); software used to prepare material for publication: *SHELXTL*.

## Supplementary Material

Crystal structure: contains datablocks I, global. DOI: 10.1107/S1600536809005583/im2093sup1.cif

Structure factors: contains datablocks I. DOI: 10.1107/S1600536809005583/im2093Isup2.hkl

Additional supplementary materials:  crystallographic information; 3D view; checkCIF report

## Figures and Tables

**Fig. 1.**
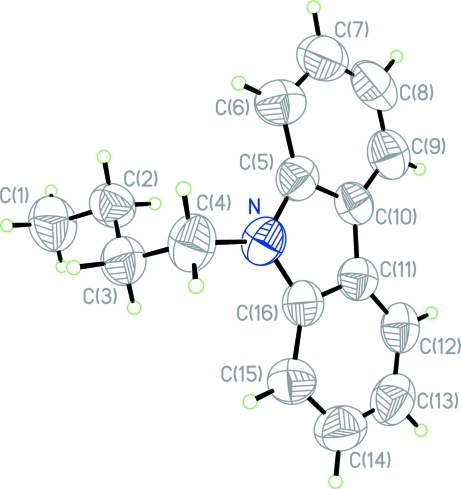
The molecular structure of (I), showing the atom-numbering scheme. Displacement ellipsoids are drawn at the 50% probability level.

## supplementary crystallographic information

### Comment

The title compound, C_16_H_17_N, is a carbazole derivative that has been designed and synthesized as a potential organic electronic device, such as OLED (Yang *et al.*, 2004). We report herein the crystal structure of the title compound, (I), which is of interest to us in the field.

The molecular structure of (I) is shown in Fig. 1. The bond lengths and angles are within normal ranges (Allen *et al.*, 1987). The tricyclic aromatic ring system is essentially planar. There are no classical hydrogen bonds observed in the crystal structure.

### Experimental

The title compound, (I), was prepared by a method reported in literature (Yang *et al.*, 2004). The crystals were obtained by dissolving (I) (0.2 g) in petroleum ether (b.p. 60–90 °C) (50 ml) and evaporating the solvent slowly at room temperature for about 3 d.

### Refinement

In the absence of significant anomalous dispersion effects, Friedel pairs were averaged. H atoms were positioned geometrically, C—H = 0.93 and 0.97 Å for aromatic and methyl H, and constrained to ride on their parent atoms, with *U*_iso_(H) = *xU*_eq_(C/O), where *x* = 1.2 for aromatic H and *x* = 1.5 for other H.

### Crystal data

**Table:**

C_16_H_17_N	*F*(000) = 480
*M**_r_* = 223.31	*D*_x_ = 1.165 Mg m^−^^3^
Orthorhombic, *P*2_1_2_1_2_1_	Mo *K*α radiation, λ = 0.71073 Å
Hall symbol: P 2ac 2ab	Cell parameters from 25 reflections
*a* = 5.544 (1) Å	θ = 9–13°
*b* = 11.276 (2) Å	µ = 0.07 mm^−^^1^
*c* = 20.369 (4) Å	*T* = 298 K
*V* = 1273.4 (4) Å^3^	Needle, colourless
*Z* = 4	0.30 × 0.20 × 0.10 mm

### Data collection

**Table:**

Enraf–Nonius CAD-4 diffractometer	1500 reflections with *I* > 2σ(*I*)
Radiation source: fine-focus sealed tube	*R*_int_ = 0.062
graphite	θ_max_ = 25.3°, θ_min_ = 2.0°
ω/2θ scans	*h* = 0→6
Absorption correction: ψ scan (North *et al.*, 1968)	*k* = 0→13
*T*_min_ = 0.980, *T*_max_ = 0.993	*l* = −24→24
2671 measured reflections	3 standard reflections every 200 reflections
1372 independent reflections	intensity decay: 1%

### Refinement

**Table:**

Refinement on *F*^2^	Primary atom site location: structure-invariant direct methods
Least-squares matrix: full	Secondary atom site location: difference Fourier map
*R*[*F*^2^ > 2σ(*F*^2^)] = 0.059	Hydrogen site location: inferred from neighbouring sites
*wR*(*F*^2^) = 0.147	H-atom parameters constrained
*S* = 1.00	*w* = 1/[σ^2^(*F*_o_^2^) + (0.06*P*)^2^ + 0.13*P*] where *P* = (*F*_o_^2^ + 2*F*_c_^2^)/3
1372 reflections	(Δ/σ)_max_ < 0.001
154 parameters	Δρ_max_ = 0.17 e Å^−^^3^
0 restraints	Δρ_min_ = −0.14 e Å^−^^3^

### Special details

**Table:**

Geometry. All e.s.d.'s (except the e.s.d. in the dihedral angle between two l.s. planes) are estimated using the full covariance matrix. The cell e.s.d.'s are taken into account individually in the estimation of e.s.d.'s in distances, angles and torsion angles; correlations between e.s.d.'s in cell parameters are only used when they are defined by crystal symmetry. An approximate (isotropic) treatment of cell e.s.d.'s is used for estimating e.s.d.'s involving l.s. planes.
Refinement. Refinement of *F*^2^ against ALL reflections. The weighted *R*-factor *wR* and goodness of fit *S* are based on *F*^2^, conventional *R*-factors *R* are based on *F*, with *F* set to zero for negative *F*^2^. The threshold expression of *F*^2^ > σ(*F*^2^) is used only for calculating *R*-factors(gt) *etc*. and is not relevant to the choice of reflections for refinement. *R*-factors based on *F*^2^ are statistically about twice as large as those based on *F*, and *R*- factors based on ALL data will be even larger.

### Fractional atomic coordinates and isotropic or equivalent isotropic displacement parameters (Å^2^)

**Table:**

	*x*	*y*	*z*	*U*_iso_*/*U*_eq_	
N	0.1136 (6)	0.1561 (2)	0.16013 (14)	0.0639 (8)	
C1	−0.0693 (11)	−0.1920 (4)	0.0678 (2)	0.1075 (18)	
H1A	−0.2201	−0.2294	0.0782	0.161*	
H1B	0.0570	−0.2502	0.0680	0.161*	
H1C	−0.0796	−0.1563	0.0251	0.161*	
C2	−0.0144 (8)	−0.0965 (3)	0.1188 (2)	0.0770 (12)	
H2A	−0.1447	−0.0391	0.1191	0.092*	
H2B	−0.0074	−0.1329	0.1619	0.092*	
C3	0.2163 (8)	−0.0341 (3)	0.10593 (19)	0.0748 (12)	
H3A	0.2051	0.0050	0.0636	0.090*	
H3B	0.3441	−0.0926	0.1030	0.090*	
C4	0.2869 (8)	0.0583 (3)	0.1576 (2)	0.0784 (12)	
H4A	0.2952	0.0205	0.2003	0.094*	
H4B	0.4456	0.0894	0.1474	0.094*	
C5	−0.0714 (7)	0.1694 (3)	0.20522 (18)	0.0642 (9)	
C6	−0.1348 (8)	0.0987 (4)	0.25810 (19)	0.0764 (12)	
H6A	−0.0507	0.0294	0.2676	0.092*	
C7	−0.3244 (11)	0.1343 (4)	0.2957 (2)	0.0937 (15)	
H7A	−0.3660	0.0899	0.3325	0.112*	
C8	−0.4570 (10)	0.2346 (4)	0.2807 (2)	0.0953 (15)	
H8A	−0.5891	0.2548	0.3065	0.114*	
C9	−0.3964 (8)	0.3046 (4)	0.2283 (2)	0.0810 (12)	
H9A	−0.4878	0.3713	0.2182	0.097*	
C10	−0.1954 (7)	0.2748 (3)	0.18976 (18)	0.0620 (9)	
C11	−0.0777 (7)	0.3272 (3)	0.13483 (17)	0.0621 (9)	
C12	−0.1083 (9)	0.4315 (3)	0.0996 (2)	0.0741 (12)	
H12A	−0.2352	0.4824	0.1095	0.089*	
C13	0.0510 (11)	0.4591 (3)	0.0499 (2)	0.0903 (15)	
H13A	0.0320	0.5295	0.0266	0.108*	
C14	0.2388 (11)	0.3834 (4)	0.0341 (2)	0.0879 (14)	
H14A	0.3439	0.4040	0.0004	0.105*	
C15	0.2736 (9)	0.2782 (3)	0.06719 (19)	0.0759 (11)	
H15A	0.3981	0.2269	0.0559	0.091*	
C16	0.1142 (7)	0.2517 (3)	0.11837 (18)	0.0621 (9)	

### Atomic displacement parameters (Å^2^)

**Table:**

	*U*^11^	*U*^22^	*U*^33^	*U*^12^	*U*^13^	*U*^23^
N	0.0568 (19)	0.0591 (15)	0.0759 (19)	0.0061 (15)	−0.0073 (18)	0.0044 (14)
C1	0.124 (5)	0.098 (3)	0.100 (3)	−0.004 (4)	−0.009 (4)	−0.027 (3)
C2	0.069 (3)	0.083 (2)	0.079 (3)	−0.001 (2)	0.012 (3)	−0.003 (2)
C3	0.072 (3)	0.069 (2)	0.083 (3)	0.009 (2)	0.005 (2)	0.002 (2)
C4	0.071 (3)	0.068 (2)	0.097 (3)	0.016 (2)	−0.007 (3)	0.003 (2)
C5	0.067 (2)	0.0624 (19)	0.063 (2)	−0.0042 (19)	−0.008 (2)	−0.0021 (17)
C6	0.074 (3)	0.086 (3)	0.070 (2)	−0.010 (2)	−0.007 (3)	0.005 (2)
C7	0.106 (4)	0.102 (3)	0.073 (3)	−0.017 (3)	0.011 (3)	−0.005 (2)
C8	0.090 (4)	0.117 (3)	0.079 (3)	−0.015 (3)	0.015 (3)	−0.035 (3)
C9	0.069 (3)	0.087 (3)	0.087 (3)	0.003 (2)	−0.004 (3)	−0.018 (2)
C10	0.061 (2)	0.066 (2)	0.059 (2)	0.0000 (18)	−0.002 (2)	−0.0108 (17)
C11	0.062 (2)	0.0638 (18)	0.060 (2)	−0.002 (2)	−0.009 (2)	−0.0041 (16)
C12	0.082 (3)	0.061 (2)	0.080 (3)	0.010 (2)	−0.017 (3)	−0.0081 (19)
C13	0.120 (4)	0.069 (2)	0.081 (3)	−0.006 (3)	−0.017 (3)	0.005 (2)
C14	0.100 (4)	0.091 (3)	0.072 (3)	−0.017 (3)	0.004 (3)	0.008 (2)
C15	0.070 (3)	0.077 (2)	0.081 (3)	−0.006 (2)	0.010 (2)	−0.002 (2)
C16	0.061 (2)	0.0595 (17)	0.066 (2)	0.0048 (19)	−0.004 (2)	−0.0030 (17)

### Geometric parameters (Å, °)

**Table:**

N—C16	1.373 (4)	C6—H6A	0.9300
N—C5	1.385 (4)	C7—C8	1.384 (6)
N—C4	1.463 (4)	C7—H7A	0.9300
C1—C2	1.527 (5)	C8—C9	1.369 (5)
C1—H1A	0.9600	C8—H8A	0.9300
C1—H1B	0.9600	C9—C10	1.403 (5)
C1—H1C	0.9600	C9—H9A	0.9300
C2—C3	1.483 (6)	C10—C11	1.423 (5)
C2—H2A	0.9700	C11—C12	1.388 (4)
C2—H2B	0.9700	C11—C16	1.403 (5)
C3—C4	1.532 (5)	C12—C13	1.380 (6)
C3—H3A	0.9700	C12—H12A	0.9300
C3—H3B	0.9700	C13—C14	1.384 (6)
C4—H4A	0.9700	C13—H13A	0.9300
C4—H4B	0.9700	C14—C15	1.378 (5)
C5—C6	1.385 (5)	C14—H14A	0.9300
C5—C10	1.409 (4)	C15—C16	1.399 (5)
C6—C7	1.361 (6)	C15—H15A	0.9300
			
C16—N—C5	109.1 (3)	C5—C6—H6A	121.2
C16—N—C4	124.6 (3)	C6—C7—C8	121.8 (4)
C5—N—C4	126.2 (3)	C6—C7—H7A	119.1
C2—C1—H1A	109.5	C8—C7—H7A	119.1
C2—C1—H1B	109.5	C9—C8—C7	120.9 (5)
H1A—C1—H1B	109.5	C9—C8—H8A	119.6
C2—C1—H1C	109.5	C7—C8—H8A	119.6
H1A—C1—H1C	109.5	C8—C9—C10	119.5 (4)
H1B—C1—H1C	109.5	C8—C9—H9A	120.2
C3—C2—C1	112.7 (4)	C10—C9—H9A	120.2
C3—C2—H2A	109.0	C9—C10—C5	117.7 (4)
C1—C2—H2A	109.0	C9—C10—C11	134.8 (4)
C3—C2—H2B	109.0	C5—C10—C11	107.6 (3)
C1—C2—H2B	109.0	C12—C11—C16	118.9 (4)
H2A—C2—H2B	107.8	C12—C11—C10	134.5 (4)
C2—C3—C4	114.9 (4)	C16—C11—C10	106.5 (3)
C2—C3—H3A	108.5	C13—C12—C11	119.5 (4)
C4—C3—H3A	108.5	C13—C12—H12A	120.3
C2—C3—H3B	108.5	C11—C12—H12A	120.3
C4—C3—H3B	108.5	C12—C13—C14	120.9 (4)
H3A—C3—H3B	107.5	C12—C13—H13A	119.6
N—C4—C3	111.6 (3)	C14—C13—H13A	119.6
N—C4—H4A	109.3	C15—C14—C13	121.5 (4)
C3—C4—H4A	109.3	C15—C14—H14A	119.2
N—C4—H4B	109.3	C13—C14—H14A	119.2
C3—C4—H4B	109.3	C14—C15—C16	117.4 (4)
H4A—C4—H4B	108.0	C14—C15—H15A	121.3
C6—C5—N	129.9 (4)	C16—C15—H15A	121.3
C6—C5—C10	122.4 (4)	N—C16—C15	129.1 (4)
N—C5—C10	107.7 (3)	N—C16—C11	109.1 (3)
C7—C6—C5	117.7 (4)	C15—C16—C11	121.8 (3)
C7—C6—H6A	121.2		
			
C1—C2—C3—C4	−177.0 (3)	C9—C10—C11—C12	3.9 (7)
C16—N—C4—C3	−82.4 (4)	C5—C10—C11—C12	−176.8 (4)
C5—N—C4—C3	99.4 (4)	C9—C10—C11—C16	−179.1 (4)
C2—C3—C4—N	−64.0 (4)	C5—C10—C11—C16	0.2 (4)
C16—N—C5—C6	−176.8 (4)	C16—C11—C12—C13	−0.6 (5)
C4—N—C5—C6	1.6 (6)	C10—C11—C12—C13	176.1 (4)
C16—N—C5—C10	2.0 (4)	C11—C12—C13—C14	0.8 (6)
C4—N—C5—C10	−179.6 (3)	C12—C13—C14—C15	0.1 (7)
N—C5—C6—C7	178.7 (4)	C13—C14—C15—C16	−1.2 (6)
C10—C5—C6—C7	0.1 (5)	C5—N—C16—C15	177.2 (4)
C5—C6—C7—C8	2.6 (6)	C4—N—C16—C15	−1.2 (6)
C6—C7—C8—C9	−2.3 (7)	C5—N—C16—C11	−1.9 (4)
C7—C8—C9—C10	−0.7 (6)	C4—N—C16—C11	179.7 (3)
C8—C9—C10—C5	3.2 (5)	C14—C15—C16—N	−177.5 (4)
C8—C9—C10—C11	−177.5 (4)	C14—C15—C16—C11	1.5 (5)
C6—C5—C10—C9	−3.0 (5)	C12—C11—C16—N	178.6 (3)
N—C5—C10—C9	178.1 (3)	C10—C11—C16—N	1.0 (4)
C6—C5—C10—C11	177.6 (3)	C12—C11—C16—C15	−0.6 (5)
N—C5—C10—C11	−1.3 (4)	C10—C11—C16—C15	−178.1 (3)
